# Course of Self-Reported Dysphagia, Voice Impairment and Pain in Head and Neck Cancer Survivors

**DOI:** 10.3390/biology10020144

**Published:** 2021-02-11

**Authors:** Veit Zebralla, Susanne Wiegand, Andreas Dietz, Gunnar Wichmann, Thomas Neumuth, Anja Mehnert-Theuerkauf, Andreas Hinz

**Affiliations:** 1 Department of Otolaryngology, Head and Neck Surgery, University of Leipzig, 04103 Leipzig, Germany; susanne.wiegand@medizin.uni-leipzig.de (S.W.); andreas.dietz@medizin.uni-leipzig.de (A.D.); gunnar.wichmann@medizin.uni-leipzig.de (G.W.); 2 Innovation Center Computer Assisted Surgery (ICCAS), University of Leipzig, 04103 Leipzig, Germany; thomas.neumuth@medizin.uni-leipzig.de; 3 Department of Medical Psychology and Medical Sociology, University of Leipzig, 04103 Leipzig, Germany; anja.mehnert@medizin.uni-leipzig.de (A.M.-T.); andreas.hinz@medizin.uni-leipzig.de (A.H.)

**Keywords:** head and neck cancer, HNC, survivor, PRO, dysphagia, aftercare, OncoFunction

## Abstract

**Simple Summary:**

Patients with head and neck cancer often suffer from multiple and severe functional impairments. Swallowing, voice impairment and pain are often mentioned as mostly relevant for patients’ quality of life after treatment. The course of these specific functional impairments and related problems are not sufficiently observed. In our retrospective single-center cohort analysis of “real-world data”, collected in daily routine practice, we present data regarding the patient-reported outcome parameters of swallowing and voice problems and pain. Independent of tumor site and treatment regimen, patients reported less problems over time. Nevertheless, oropharyngeal tumors led to significantly more self-reported swallowing problems, while patients with larynx tumors more often had patient-perceived voice impairments. In addition, other clinical and sociodemographic variables had an impact on patient-reported function. The acquisition of patient-reported outcome data is valuable and a sufficient way to explore patients’ problems in a better manner. These data can help to improve patient care.

**Abstract:**

Background: Head and neck cancer (HNC)-specific symptoms have a substantial impact on health-related quality of life. The aim of this study was to determine whether self-reported dysphagia, voice problems and pain of HNC patients changed over time and whether specific clinical or sociodemographic variables were associated with these symptoms. Methods: HNC patients (*n* = 299) in an outpatient setting answered questionnaires (Eating Assessment Tool-10; questions from the EORTC QLQ-C30 and EORTC H&N35) on dysphagia, voice problems and pain, collected with the software “OncoFunction” at three different timepoints (t1–t3) after diagnosis. The mean score changes from t1 to t3 were expressed in terms of effect sizes *d*. The impact of sociodemographic and clinical factors on the course of the variables was tested with multivariate analyses of variance. Results: Dysphagia, voice impairment and pain in HNC survivors significantly improved over a period of approximately 14 months after diagnosis. Tumor site, stage, treatment modality, occupational state and ECOG state were significantly correlated with self-reported functional outcome. The pain level of the HNC patients was rather low. Conclusions: Patients suffer from functional impairments after HNC treatment, but an improvement in self-reported symptoms could be demonstrated within this time period.

## 1. Introduction

Recent advancements in the detection and treatment of head and neck cancer (HNC) and the changing epidemiology, especially due to the rise in HPV-positive oropharynx cancer, have resulted in an increase in HNC survivors. The critical role of the head and neck in function as well as the partly complex treatment of HNC places survivors at high risk for HNC-specific symptoms that have a substantial impact on health-related quality of life. Tumor growth and infiltration of functional relevant tissue as well as consequences of surgery or radiation fibrosis often lead to severe functional impairments. Dysphagia, voice impairment and pain are typical symptoms that are often associated with social and psychological problems. These symptoms cause frustration and embarrassment, being common in the long term in HNC patients. Therefore, the assessment of patient-perceived dysphagia, voice problems and pain during and after HNC treatment is fundamental to measure the consequences of treatment over time. Studies have highlighted the importance of long-term swallowing and voice impairment, with a small number of studies specifically focusing on these issues [1,2,3,4,5,6]. Especially swallowing is strongly associated with impaired quality of life, which has been demonstrated in several studies [7,8,9]. Moreover, dysphagia and pain are independent risk factors for worse survival [10,11,12,13,14]. However, few longitudinal studies analyzed the follow-up of HNC-specific symptoms. Therefore, the improvement of physical functioning and self-reported impairments over time is not well studied in HNC patients. However, information about the course of functional deficiencies is important to explain the treatment and possible impairments after therapy and improve the therapy adherence of HNC patients.

As evaluation and treatment of short- and long-term side effects are important elements of comprehensive cancer survivorship care, we aimed to analyze the course of the self-reported symptoms dysphagia, voice problems and pain and to further investigate the temporal stability of these symptoms. We further aimed to analyze the impact of sociodemographic and clinical factors on the course of these symptoms.

## 2. Materials and Methods

### 2.1. Patients

Patients’ data were obtained from the software “OncoFunction”. This database contains data from outpatients with head and neck cancer seen for regular follow-up appointments who were older than 18 years and have provided informed consent. This retrospective chart review study involving human participants was in accordance with the ethical standards of the institutional and national research committee and with the 1964 Helsinki Declaration and its later amendments or comparable ethical standards.

We included only those patients who were able to read and complete questionnaires on a tablet computer. Collection of the surveys began in June 2013. Every HNC patient in our outpatient setting used a touch-screen tablet computer to answer questionnaires focusing on HNC patients’ problems such as pain and swallowing, voice, breathing and psychosocial problems. They were asked to return the tablet to the nurses before they were called to see the physician. The staff were trained to assist the patients with the tablet computers circulated in the waiting room. A summary of the current responses along with prior responses was provided to the physician before the consultation. The usability of the system was demonstrated before [15]. Presently, there are approximately 1200 head and neck cancer patients in the database “OncoFunction”. For this analysis, we only considered those HNC patients who had complete data at the first three follow-up visits after diagnosis and treatment of HNC (t1–t3) in at least one of the three measures (Eating Assessment Tool-10 (EAT-10), voice problems and pain). Complete data means that data from t1 to t3 were available. The follow-up schedule was based on National Comprehensive Cancer Network (NCCN) guidelines. In the first year, visits were conducted every 1–3 months, and in the second year, every 2–6 months.

Patients were retrospectively classified according to age, gender, occupational state, smoking status, alcohol consumption, tumor site and stage, type of treatment, presence of tracheotomy, presence of feeding tube, ECOG (Eastern Cooperative Oncology Group) state and body mass index (BMI).

The standard of care for radiotherapy is intensity-modulated radiation therapy (IMRT) in our clinic. The majority of patients received platinum-based chemotherapy concordantly to radiotherapy in the adjuvant or primary treatment setting. A minority of patients received immunotherapy with cetuximab in their treatment regimens.

### 2.2. Instruments

The Eating Assessment Tool (EAT-10) is a 10-item questionnaire addressing the main aspects of dysphagia. The EAT-10 questionnaire addresses questions regarding loss of weight, swallowing effort, pain while swallowing, coughing and meals being stuck while eating and impaired social eating. It has been shown to correlate with findings on instrumental swallowing assessments such as a fiberoptic endoscopic evaluation of swallowing (FEES) [16,17,18]. Patients rate several swallowing issues on a 5-point scale (0 = no problem, 4 = severe problem), leading to an overall score ranging from 0 to 40 points. Based on normative data from healthy volunteers, a total score of three or higher is considered abnormal.

Voice problems were analyzed using the questionnaire “European Organization for Research and Treatment of Cancer Quality of Life Questionnaire-Head and Neck 35” (EORTC QLQ-H&N35), a specific self-report questionnaire for HNC patients [19]. The patients indicate the extent to which they have experienced problems when talking to other people or talking on the telephone. The responses to these two questions are scored on a four-point scale: not at all (1); a little (2); quite a bit (3) and very much (4). The responses are converted into 0–100 scales according to the methodology of the “European Organization for Research and Treatment of Cancer Core Quality of Life Questionnaire” (EORTC QLQ-C30).

Patients were asked to rate their current pain intensity on a numeric scale ranging from 0 to 10, with 0 representing “no pain at all” and 10 representing “maximal possible pain”.

### 2.3. Statistical Analysis

The mean score changes from t1 to t3 were expressed in terms of effect sizes *d*. Pearson correlation coefficients were used to characterize the temporal stability of the variables. The impact of sociodemographic and clinical factors on the course of the variables was statistically tested with multivariate analyses of variance (ANOVAs). In these analyses, the factor time (t1, t2, t3) served as a within-subject factor, and the clinical variable was considered the independent between-subjects factor. When clinical variables were analyzed in this way, age group and gender were additionally included as covariates. All statistical calculations were performed with SPSS version 24.

## 3. Results

### 3.1. Sample Characteristics

The sample consisted of 223 male and 76 female patients, and the mean age was 60.8 ± 10.2 years (males: 61.0 ± 10.0 years, females: 60.5 ± 10.6 years). Table 1 gives further characteristics of the sample. The mean time between diagnosis and the t1 assessment was 5.6 months (SD = 2.5 months), the mean time interval between t2 and t1 was about four months (133.1 days, SD = 119 days) and the mean interval between t3 and t1 was about twice as long (260.3 days, SD = 155 days).

### 3.2. Mean Scores

Mean scores and standard deviations of the variables are given in Table 2. The effect sizes *d*(t1, t3) refer to the mean score change from t1 to t3; negative *d* values indicate a decline. *r*(1,3) indicates the correlation between the t1 and the t3 scores.

Among the 10 items of EAT-10, we observe a decline from t1 to t3 in eight items; the largest difference (*d* = 0.55) was found for the first item (weight loss), which means that the patients gained weight. Voice problems and pain significantly reduced between t1 and t3.

The coefficients of temporal stability of the three scales were between 0.46 (pain) and 0.59 (EAT-10 sum score), and the lowest stability scores were found for EAT-10 item 3 (swallowing liquids, *r*_tt_ = 0.29) and item 1 (weight loss, *r*_tt_ = 0.35).

### 3.3. Impact of Sociodemographic and Clinical Factors on Dysphagia, Voice Problems and Pain

**Figure 1:**
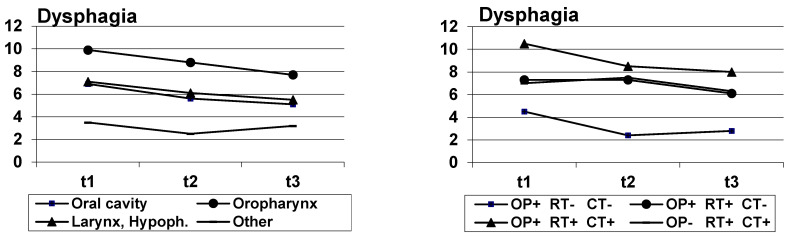
Dysphagia mean scores, broken down by diagnosis (**left**) and treatment (**right**); OP+, OP-: surgery yes/no; RT+, RT-: radiotherapy yes/no; CT+, CT-: chemotherapy yes/no.

**Figure 2:**
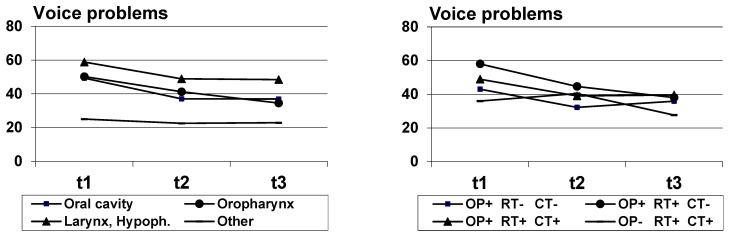
Voice problems mean scores, broken down by diagnosis (**left**) and treatment (**right**); OP+, OP-: surgery yes/no; RT+, RT-: radiotherapy yes/no; CT+, CT-: chemotherapy yes/no.

**Figure 3:**
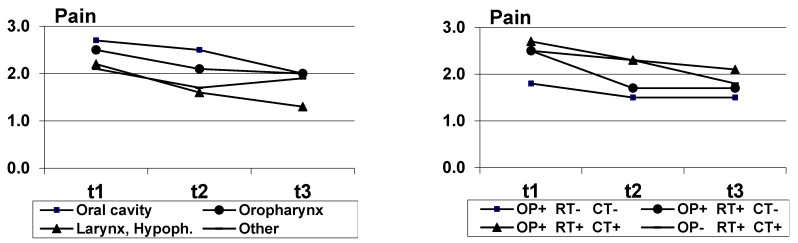
Pain mean scores, broken down by diagnosis (**left**) and treatment (**right**); OP+, OP-: surgery yes/no; RT+, RT-: radiotherapy yes/no; CT+, CT-: chemotherapy yes/no.

Figure 1, Figure 2 and Figure 3 indicate the courses of the three symptoms, broken down by diagnosis groups (left diagram) and treatment (right diagram). Since the case numbers in the treatment group “other” were low (*n* = 12), this group was not considered in the diagrams. All figures indicate a decline in the symptoms. The results of significance tests for the independent factors (diagnosis group, treatment group) and for the factor timepoint are given in Table 3, Table 4 and Table 5. The highest scores in self-reported dysphagia were observed for patients with oropharynx cancer, the strongest voice problems were reported by patients with larynx and hypopharynx cancer, and concerning pain, the patients with cancer of the oral cavity were most strongly affected. Regarding the treatment regimens, the results suggest that all presented patient groups had a substantial shift towards symptom improvement from t1 to t3. Dysphagia and pain were most often reported by patients who had trimodality treatment (surgery + radiochemotherapy) while patients who only had surgery had the lowest scores over time. Patients who were treated by surgery and radiotherapy were mostly affected by voice problems at t1; over time, voice problems improved in all treatment groups, and at t3, patients who were treated by radiochemotherapy had the lowest scores. Detailed analyses of the effects of diagnosis group, treatment and other sociodemographic and clinical variables are given in Table 3, Table 4 and Table 5.

The effect of gender and age on patient-perceived dysphagia, voice impairment or pain was mostly not significant. Patients in the age group of 60–69 years reported significantly higher dysphagia values and patients older than 70 years had the lowest dysphagia levels. Behavioral factors such as alcohol consumption or smoking did not significantly influence dysphagia and voice problems. Smokers reported more pain than non-smokers; in both groups, pain decreased over time.

Presence of metastasis and higher tumor stage did significantly influence dysphagia scores but not voice problems or pain. Patients with highest tumor stage (IV) and need of trimodality treatment (surgery + chemoradiation) reported the highest dysphagia scores over the complete follow-up. Voice problems and pain decreased from t1 to t3, independent of tumor stage or metastasis.

Regarding feeding tube and dysphagia, there was a weak relationship. Patients with feeding tubes reported higher dysphagia scores. In the same direction, patients with the need for tracheotomy reported significantly higher voice problems. Patients with a tracheal canula and feeding tube reported higher pain scores, even if not reaching significance.

Patients with better ECOG performance scores had less dysphagia and voice problems. Voice impairment significantly decreased in all ECOG groups. Patients with bad ECOG state also had higher pain levels; however, the difference was not significant. Pain levels decreased over time in all ECOG groups.

Patients with lower BMI had higher dysphagia and pain scores, but these differences did not reach significance. Patients with a BMI lower than 20 reported significantly higher voice problems from t1 to t3 and also higher dysphagia scores. Dysphagia, voice problems and pain improved over time in all BMI groups.

The presence of a feeding tube and tracheostomy (Table 6) was also examined over time. Almost two-thirds of the patients never had a feeding tube (60.4%). About 20% of all patients could abandon the feeding tube at t2 or t3, and 16.1% needed a feeding tube for the complete follow-up period. A similar picture is also seen for the presence of tracheostomy.

## 4. Discussion

This study provided data regarding the symptoms dysphagia, voice impairment and pain in head and neck cancer survivors in a short-term follow-up. The findings revealed that over a period of approximately 14 months after diagnosis of HNC, dysphagia, voice problems and pain significantly improved in our patient cohort.

Dysphagia was analyzed using the EAT-10 questionnaire, which has previously been shown to correlate with objective swallowing examinations such as videofluoroscopy and FEES and seems to be an adequate instrument for the screening for dysphagia and aspiration in daily routine practice [18,20,21]. In our patient cohort, self-reported dysphagia was significantly correlated to age, occupational state, tumor site, tumor stage, presence of metastases, treatment and ECOG performance. Young patients had significantly more swallowing problems than patients older than 70 years; however, while swallowing impairment decreased over the study period in young patients, in the patient group older than 70 years, there was no improvement. Nevertheless, patients over 70 years of age had less dysphagia at t3 than younger patients. The effect of age on swallowing function and rehabilitation potential has been reported in previous studies. For example, Wilson et al. reported that younger patients more likely report poorer swallowing results [8]. However, patient-reported swallowing performance does not correlate with objective swallowing performance in all cases [22,23]. Overall, it remains unclear if elderly HNC patients indeed show better swallowing performance than young patients.

Patients with oropharyngeal carcinomas showed higher dysphagia values at any given time than patients with other tumor sites. In the same manner, Carmignani reported significantly higher dysphagia in patients with oropharyngeal cancer compared to patients with larynx/hypopharynx cancer [1]. Additionally, patients with multimodal treatment showed higher swallowing impairment than patients with curative treatment by surgery alone. As often reported, patients with higher tumor stages often need to be treated multimodally and, so far, have a higher risk for dysphagia than patients with monomodal treatment. In our cohort, the reported values for dysphagia decreased over time, independent of tumor site and treatment regimen. This is contradictory to a study on swallowing in the first year after chemoradiotherapy for HNC, where only limited changes in the patients’ perception were reported [24].

Patients with a body mass index <20 reported the highest scores in the EAT-10 questionnaire. Insufficient supplementation could lead to weight loss and may explain the differences [25].

The presence of feeding tubes from t1 to t3 was also explored. In total, 165 patients (60.4%) reported never having used a feeding tube, and 44 patients had to have their diet with a feeding tube all the time. Compared to t1, the feeding tube was removed at t2 in 39 patients and at t3 in 55 patients. This supports the statement that the course of dysphagia allows many patients an oral diet and better swallowing-related quality of life.

The presence of tracheostomy tube also decreased over time in our patient cohort. Previous studies demonstrated that patients’ swallowing function correlates with time of decannulation, as one would expect, because only the exclusion of aspiration can prevent pneumonia and related problems [26]. In our cohort, in 39 patients, the tracheostomy tube could be permanently removed, whereas nine patients needed a re-cannulation. Furthermore, 42 patients were still tracheotomized at the end of the evaluation. Correlation of patients’ symptom monitoring with clinical examination parameters can help to improve decision making and define the best moment for decannulation or gastrostomy tube removal. Further research and studies examining these topics are needed.

Patient-reported voice impairment significantly improved over the first three follow-up consultations. The same course was described in other publications, e.g., for transoral laser cordectomy [27]. In this publication, the main improvement in voice was within the first 6 months postoperatively, and in our cohort also, the main effect occurred from t1 to t2. In total laryngectomized patients, van Sluis reported less subjective impairment of patients’ reported voice quality after 12 months, but in the objective study, a high number of patients did not achieve significant improvement [28]. Regarding their voice impairment, patients with laryngeal or hypopharyngeal carcinomas reported significantly higher values than patients with cancer of the oral cavity/oropharynx or other localizations from t1 to t3, independent of their treatment regimen (multimodal vs. monomodal). This seems comprehensible because the tumor- and treatment-related side effects directly influence the voice through modified function of the vocal fold through scarring and fibrosis [29]. Additionally, the presence of tracheotomy and feeding tubes showed negative associations but a diminishing impairment over time.

Concerning pain, there was a significant improvement over time in our HNC patient cohort. Pain decreased in all examined items. Overall, the pain level was rather low in our cohort. This seems to be contradictory to results that characterized HNC patients by significant greater pain perception compared to other tumor entities [30]. Bossi et al. reported that up to 80% of HNC patients reported pain, which cannot be confirmed by our retrospective analysis [31]. The decreasing pain level over time matches the study of Chaplin et al. [32]. In a cohort of 93 patients, the prevalence of pain decreased from 84% at diagnosis of HNC to 25% 12 months later [32]. In our cohort, the pain level differs significantly between smokers and non-smokers; smokers reported higher pain values in the numeric analogue scale.

We could not detect a gender effect regarding the examined variables. Swallowing function, voice impairment and pain improved over time in men and women, independent of their gender. The impact of behavioral factors such as tobacco and/or alcohol consumption on the analyzed symptoms remains unclear. In the presented cohort, the data were inconclusive. Occupied HNC patients had significantly lower dysphagia and less voice problems at the analyzed follow-up points of time, than non-occupied patients. Whether patients with better function and less impairment are more frequently employed or whether employment leads to better patient-reported physical function remains unclear. However, the impact of employment status and the barriers to return to work after HNC were demonstrated in several studies [33,34]. In the present study, we demonstrated that dysphagia and voice problems improved over time independently of employment status.

Concerning ECOG state, our data are inconclusive. While dysphagia and voice problems were significantly correlated with ECOG status, pain was not; however, voice impairment and pain significantly improved over time depending on the performance status. This might be due to the potential of better compensation of functional limitations during the course with a better ECOG status.

To explore the effect size of the variables of the EAT-10, voice and pain scale and to test the temporal stability, additional statistical analyses were performed. The highest effect size was shown for the question of weight loss and painful swallowing and the lowest temporal stability was shown for swallowing liquids and, again, weight loss. It has to be mentioned that the majority of questions showed high stability, and differences over time may be triggered only by a few questions. In further research, the possibility to reduce the questionnaire to some stable items could be examined.

The strength of the present study is that all of the HNC patients were assessed using standardized and structured instruments.

Nevertheless, the present study has some limitations, mainly due to its retrospective design. The reported data were self-reported, and while this has many benefits such as obtaining unfiltered patient data, a substantial bias through wrong reporting cannot be excluded. We tried to reach all patients in our aftercare consultation but may have excluded special patient groups such as illiterate patients or patients with Korsakow’s syndrome.

## 5. Conclusions

Dysphagia, voice impairment and pain in HNC survivors significantly improved over a period of approximately 14 months after diagnosis. Tumor site, stage, treatment modality, occupational state and ECOG state were significantly correlated with functional outcome. The pain level was rather low in the analyzed patient cohort. These data show the value of patient-reported outcomes collected in the daily routine practice. The illustration of postoperative courses and knowledge about “normative ranges” can help to interpret patient-reported outcome data in the future.

## Figures and Tables

**Table 1:** Patient characteristics.

	Total (*n* = 299)		Males (*n* = 223)		Females (*n* = 76)	
	*n*	%	*n*	%	*n*	%
Age Group						
18–59 years	144	48.2	104	46.6	40	52.6
60–69 years	98	32.8	78	35.0	20	26.3
≥70 years	57	19.1	41	18.4	16	21.1
Occupation						
Not occupied	219	73.2	167	74.9	52	68.4
Occupied	80	26.8	56	25.1	24	31.6
Alcohol consumption *						
No	225	75.5	156	70.0	69	92.0
Yes	73	24.5	67	30.0	6	8.0
Tobacco consumption *						
No	218	73.2	158	71.3	59	78.7
Yes	80	26.8	64	28.7	16	21.3
Tumor group						
Oral cavity	47	15.7	33	14.8	14	18.4
Oropharynx	115	38.5	84	37.7	31	40.8
Larynx, Hypopharynx	85	28.4	74	33.2	11	14.5
Other	52	17.4	32	14.3	20	26.3
Tumor stage *						
I	53	19.1	37	17.7	16	23.5
II	33	11.9	27	12.9	6	8.8
III	51	18.4	37	17.7	14	20.6
IV	140	50.5	108	51.7	32	47.1
Treatment group						
1: OP+ RT- CT-	71	23.7	48	21.5	23	30.3
2: OP+ RT+ CT-	83	27.8	69	30.9	14	18.4
3: OP+ RT+ CT+	98	32.8	73	32.7	25	32.9
4: OP- RT+ CT+	39	13.0	26	11.7	13	17.1
5: Other	8	2.7	7	3.1	1	1.3
Metastases						
No	157	52.5	113	50.7	44	57.9
Yes	142	47.5	110	49.3	32	42.1
Tracheotomy						
No	218	70.9	151	67.7	61	80.3
Yes	87	29.1	72	32.3	15	19.7
Feeding tube						
No	186	62.8	135	61.1	51	68.0
Yes	110	37.2	86	38.9	24	32.0
ECOG performance *						
0	69	32.4	47	31.1	22	35.5
1	116	54.5	85	56.3	31	50.0
2–4	28	13.1	19	12.6	9	14.5
Body Mass Index						
<20 kg/m²	50	16.7	32	14.3	18	23.7
20–<25 kg/m²	148	49.5	115	51.6	33	43.4
25–<30 kg/m²	75	25.1	58	26.0	17	22.4
≥30 kg/m²	26	8.7	18	8.1	8	10.5

OP+, OP-: surgery yes/no; RT+, RT-: radiotherapy yes/no; CT+, CT-: chemotherapy yes/no, * missing data not reported.

**Table 2:** Mean scores for the three measurement points, effect sizes for the t1–t3 comparison and correlations between t1 and t3.

	t1		t2		t3		*d*(t1, t3)	Sign.	*r*(t1, t3)	Sign.
	M	(SD)	M	(SD)	M	(SD)				
EAT-10 (item range 0–4)										
Item 1. Weight loss	0.90	(1.12)	0.43	(0.82)	0.37	(0.81)	0.55	***	0.35	***
Item 2. Problems with meals	1.18	(1.46)	0.92	(1.28)	0.84	(1.27)	0.25	***	0.57	***
Item 3. Swallowing liquids	0.47	(0.88)	0.46	(0.84)	0.37	(0.70)	0.13	n.s.	0.29	***
Item 4. Swallowing solids	1.07	(1.23)	0.92	(1.19)	0.91	(1.19)	0.13	*	0.60	***
Item 5. Swallowing tablets	0.71	(1.17)	0.70	(1.07)	0.71	(1.09)	0.00	n.s.	0.42	***
Item 6. Swallowing painful	0.52	(0.85)	0.42	(0.78)	0.29	(0.63)	0.31	***	0.37	***
Item 7. Reduced pleasure to eat	1.00	(1.28)	0.80	(1.13)	0.73	(1.21)	0.22	**	0.57	***
Item 8. Food sticks in throat	0.29	(0.74)	0.33	(0.73)	0.37	(0.87)	−0.10	n.s.	0.54	***
Item 9. Coughing when eating	0.56	(0.93)	0.66	(0.98)	0.63	(1.02)	−0.07	n.s.	0.53	***
Item 10. Swallowing stressful	0.76	(1.00)	0.70	(1.00)	0.62	(1.03)	0.14	*	0.52	***
EAT-10 sum score (range 0–40)	7.47	(8.12)	6.41	(7.87)	5.85	(7.86)	0.20	**	0.59	***
Voice problems (range 0–100)	48.4	(35.7)	39.8	(33.3)	37.0	(33.7)	0.33	***	0.50	***
Pain (range 0–10)	2.40	(2.29)	1.94	(2.06)	1.81	(2.19)	0.26	***	0.46	***

*d*(t1, t3): Effect size for the comparison between t1 and t3; *r*(t1, t3): correlation between the t1 and t3 scores. *: *p* < 0.05; **: *p* < 0.01; ***: *p* < 0.001; n.s.: not significant.

**Table 3:** Dysphagia: Mean scores depending on sociodemographic and clinical variables.

	*n*	t1		t2		t3		Significance		
		M	(SD)	M	(SD)	M	(SD)	Main Effect	Time	Interaction
Gender								0.966	**0.020**	0.439
Males	164	7.4	(8.2)	6.5	(8.1)	5.7	(7.9)			
Females	52	7.6	(7.8)	6.1	(7.1)	6.2	(7.9)			
Age group								**0.034**	0.147	0.079
≤59 years	113	7.9	(7.9)	5.9	(7.2)	5.4	(7.2)			
60–69 years	63	8.7	(9.3)	8.3	(9.0)	7.5	(8.7)			
≥70 years	40	4.4	(6.1)	4.7	(7.5)	4.6	(7.9)			
Occupational state								**0.005**	**0.008**	0.634
Not occupied	147	8.3	(8.6)	7.3	(8.4)	6.6	(8.4)			
Occupied	69	5.8	(6.8)	4.7	(6.6)	4.2	(6.4)			
Alcohol consumption								0.241	**0.013**	0.828
No	157	7.8	(8.3)	6.7	(8.1)	6.2	(8.2)			
Yes	59	6.6	(7.5)	5.6	(7.2)	4.9	(7.0)			
Tobacco consumption								0.995	**0.012**	0.933
No	157	7.4	(8.0)	6.4	(7.8)	5.9	(8.0)			
Yes	59	7.7	(8.4)	6.5	(8.1)	5.6	(7.6)			
Tumor								**0.001**	**0.021**	0.571
Oral cavity	35	6.9	(7.9)	5.6	(7.8)	5.1	(8.5)			
Oropharynx	80	9.9	(7.7)	8.8	(7.8)	7.7	(8.4)			
Larynx, Hypopharynx	64	7.1	(8.9)	6.1	(8.4)	5.5	(7.5)			
Other	37	3.5	(5.9)	2.5	(5.0)	3.2	(5.6)			
Tumor stage								**0.002**	0.120	**0.035**
I	45	4.1	(7.9)	2.6	(6.0)	2.7	(6.1)			
II	27	4.9	(5.8)	7.4	(9.2)	5.4	(7.3)			
III	35	7.0	(6.8)	7.2	(8.5)	6.9	(9.5)			
IV	94	10.1	(8.7)	7.8	(7.8)	7.1	(8.0)			
Treatment group								**0.001**	**0.008**	0.606
1: OP+ RT- CT-	59	4.5	(7.6)	2.4	(5.3)	2.8	(5.7)			
2: OP+ RT+ CT-	62	7.3	(7.8)	7.3	(8.0)	6.1	(7.5)			
3: OP+ RT+ CT+	66	10.5	(8.4)	8.5	(8.3)	8.0	(8.5)			
4: OP- RT+ CT+	23	7.0	(7.3)	7.5	(7.6)	6.3	(8.1)			
Metastases								**0.001**	**0.003**	0.721
No	116	6.0	(8.1)	4.7	(6.9)	4.6	(7.4)			
Yes	100	9.1	(7.8)	8.4	(8.5)	7.3	(8.1)			
Tracheotomy								0.418	**0.005**	0.286
No	171	7.2	(8.4)	6.4	(8.2)	5.5	(7.9)			
Yes	45	8.4	(6.9)	6.5	(6.8)	7.3	(7.5)			
Feeding tube								0.054	**0.001**	0.295
No	165	6.7	(8.0)	6.0	(8.1)	5.4	(8.0)			
Yes	51	10.0	(8.1)	7.9	(6.9)	7.2	(7.3)			
ECOG performance								**<0.001**	0.511	0.168
0	59	4.1	(6.3)	3.7	(6.6)	3.2	(6.6)			
1	83	9.7	(8.5)	8.1	(8.1)	7.2	(7.3)			
2–4	9	7.8	(8.5)	6.8	(8.4)	6.0	(7.8)			
BMI								0.103	**0.021**	0.619
<20 kg/m²	27	9.9	(8.7)	8.5	(8.5)	8.4	(9.6)			
20–<25 kg/m²	109	8.3	(8.9)	6.6	(7.7)	6.3	(8.3)			
25–<30 kg/m²	57	5.8	(6.1)	6.2	(8.5)	4.6	(6.4)			
≥30 kg/m²	23	5.0	(7.1)	3.1	(5.5)	3.7	(5.8)			

M: Mean; SD: Standard deviation; Main effect: ANOVA effect of the independent variable listed in the left column; Time: effect of the timepoint (t1, t2, t3); Interaction: interaction between the independent variable and the factor timepoint.

**Table 4:** Voice problems: Mean scores depending on sociodemographic and clinical variables.

	*n*	t1		t2		t3		Significance		
		M	(SD)	M	(SD)	M	(SD)	Main Effect	Time	Interaction
Gender								0.781	**<0.001**	0.745
Males	212	48.0	(35.6)	39.0	(33.3)	37.7	(34.7)			
Females	72	49.5	(36.3)	42.1	(33.4)	34.7	(30.9)			
Age group								0.681	**<0.001**	0.166
≤59 years	138	47.6	(33.1)	40.6	(32.0)	36.8	(32.9)			
60–69 years	94	50.2	(36.7)	42.7	(32.8)	38.5	(33.9)			
≥70 years	52	47.4	(40.7)	31.4	(37.1)	34.6	(36.1)			
Occupational state								**0.001**	**<0.001**	0.336
Not occupied	208	52.3	(36.2)	42.7	(34.2)	41.1	(34.7)			
Occupied	76	37.7	(32.1)	32.7	(29.9)	25.7	(27.9)			
Alcohol consumption								0.162	**<0.001**	0.766
No	212	50.6	(35.6)	40.9	(32.8)	38.5	(34.0)			
Yes	72	42.1	(35.4)	36.8	(34.6)	32.4	(32.6)			
Tobacco consumption								0.654	**<0.001**	0.196
No	206	49.3	(35.6)	39.1	(33.0)	38.2	(33.7)			
Yes	78	46.2	(36.0)	41.5	(34.3)	33.8	(33.7)			
Tumor								**<0.001**	**<0.001**	0.424
Oral cavity	47	49.3	(33.0)	37.0	(31.5)	36.9	(33.3)			
Oropharynx	110	50.2	(34.6)	41.2	(32.5)	34.5	(34.1)			
Larynx, Hypopharynx	81	58.8	(33.2)	48.9	(36.1)	48.4	(33.3)			
Other	46	25.0	(35.4)	22.5	(27.4)	22.8	(28.0)			
Tumor stage								0.065	**<0.001**	0.140
I	50	40	(33.3)	28.3	(30.3)	31.7	(31.1)			
II	31	47.3	(39.0)	47.0	(37.4)	40.3	(33.0)			
III	49	42.2	(32.3)	42.0	(30.4)	30.3	(32.8)			
IV	133	54.6	(35.8)	41.5	(34.3)	41.1	(34.9)			
Treatment group								0.104	**<0.001**	0.003
1: OP+ RT- CT-	67	43.0	(36.4)	32.2	(34.5)	35.8	(33.0)			
2: OP+ RT+ CT-	79	58.0	(35.4)	44.6	(33.9)	38.0	(34.8)			
3: OP+ RT+ CT+	92	48.9	(32.7)	39.0	(31.7)	39.5	(33.8)			
4: OP- RT+ CT+	38	36.0	(36.9)	40.4	(32.3)	27.6	(30.3)			
Metastases								0.466	**<0.001**	0.807
No	147	45.7	(35.7)	39.0	(33.3)	35.8	(34.1)			
Yes	137	51.3	(35.5)	40.7	(33.3)	38.2	(33.4)			
Tracheotomy								**<0.001**	**<0.001**	0.006
No	202	40.3	(34.1)	35.0	(31.4)	29.3	(30.3)			
Yes	82	68.5	(31.4)	50.9	(35.1)	55.9	(34.5)			
Feeding tube								**0.003**	**<0.001**	0.142
No	178	42.0	(35.0)	37.3	(32.3)	32.9	(32.8)			
Yes	104	59.3	(34.3)	44.3	(34.5)	43.7	(33.8)			
ECOG performance								**<0.001**	**<0.001**	0.568
0	67	38.6	(34.6)	30.1	(31.3)	24.9	(29.5)			
1	110	50.2	(33.3)	45.0	(33.1)	41.7	(32.9)			
2–4	25	74.0	(28.1)	60.5	(36.5)	52.7	(38.4)			
BMI								**0.017**	**<0.001**	0.196
<20 kg/m²	49	55.8	(35.3)	52.7	(33.3)	41.2	(33.7)			
20–<25 kg/m²	142	44.8	(35.6)	36.6	(31.0)	36.0	(33.2)			
25–<30 kg/m²	68	50.7	(34.8)	44.6	(35.2)	39.5	(35.3)			
≥30 kg/m²	25	48.0	(38.3)	20.5	(30.8)	27.3	(32.2)			

M: Mean; SD: Standard deviation; Main effect: ANOVA effect of the independent variable listed in the left column; Time: effect of the time point (t1, t2, t3); Interaction: interaction between the independent variable and the factor time point.

**Table 5:** Pain (*n* = 294): Mean scores depending on sociodemographic and clinical variables.

	*n*	t1		t2		t3		Significance		
		M	(SD)	M	(SD)	M	(SD)	Main Effect	Time	Interaction
Gender								0.593	**0.001**	0.518
Males	218	2.38	(2.24)	1.95	(2.14)	1.76	(2.27)			
Females	76	2.46	(2.44)	1.93	(1.80)	1.95	(1.95)			
Age group								0.072	**<0.001**	0.817
≤59 years	142	2.61	(2.29)	2.24	(2.16)	2.08	(2.27)			
60–69 years	96	2.42	(2.27)	1.67	(1.89)	1.65	(2.15)			
≥70 years	56	1.86	(2.28)	1.60	(2.00)	1.39	(2.00)			
Occupational state								0.249	**<0.001**	0.472
Not occupied	215	2.45	(2.37)	2.01	(2.17)	1.95	(2.35)			
Occupied	79	2.28	(2.06)	1.78	(1.76)	1.43	(1.62)			
Alcohol consumption								0.510	**<0.001**	0.330
No	221	2.42	(2.27)	2.00	(2.06)	1.91	(2.29)			
Yes	72	2.39	(2.36)	1.80	(2.06)	1.50	(1.85)			
Tobacco consumption								**0.024**	**0.002**	0.530
No	213	2.32	(2.21)	1.77	(1.89)	1.62	(2.07)			
Yes	80	2.65	(2.50)	2.42	(2.40)	2.32	(2.44)			
Tumor								0.226	**<0.001**	0.577
Oral cavity	47	2.66	(2.34)	2.46	(2.27)	1.96	(2.33)			
Oropharynx	115	2.53	(2.11)	2.06	(2.05)	2.03	(2.18)			
Larynx, Hypopharynx	82	2.23	(2.39)	1.64	(2.10)	1.33	(1.94)			
Other	50	2.14	(2.49)	1.73	(1.75)	1.94	(2.40)			
Tumor stage								0.211	**0.004**	0.443
I	52	1.62	(1.94)	1.65	(1.90)	1.42	(1.87)			
II	32	1.84	(1.82)	1.68	(2.24)	1.62	(1.83)			
III	49	2.45	(2.48)	2.07	(2.19)	1.88	(2.32)			
IV	140	2.76	(2.30)	2.05	(2.06)	1.79	(2.16)			
Treatment group								0.083	**<0.001**	0.708
1: OP+ RT- CT-	69	1.81	(2.11)	1.48	(1.83)	1.48	(1.86)			
2: OP+ RT+ CT-	80	2.51	(2.42)	1.74	(2.03)	1.74	(2.19)			
3: OP+ RT+ CT+	98	2.68	(2.28)	2.27	(2.23)	2.13	(2.37)			
4: OP- RT+ CT+	39	2.51	(2.32)	2.29	(1.87)	1.77	(2.21)			
Metastases								0.123	**<0.001**	0.438
No	152	2.25	(2.36)	1.73	(1.95)	1.57	(1.97)			
Yes	142	2.56	(2.21)	2.18	(2.16)	2.06	(2.39)			
Tracheotomy								0.464	**<0.001**	0.095
No	208	2.32	(2.21)	1.98	(2.06)	1.66	(2.08)			
Yes	86	2.59	(2.48)	1.85	(2.07)	2.16	(2.41)			
Feeding tube								0.074	**<0.001**	**0.045**
No	184	2.08	(2.01)	1.93	(2.08)	1.59	(2.00)			
Yes	109	2.97	(2.61)	1.99	(2.02)	2.18	(2.45)			
ECOG performance								0.107	**0.001**	0.118
0	67	1.91	(2.04)	1.84	(1.90)	1.46	(1.96)			
1	115	2.16	(2.20)	1.79	(1.89)	1.89	(2.05)			
2–4	27	3.78	(2.56)	2.48	(2.19)	2.19	(2.42)			
BMI								0.290	**0.002**	0.703
<20 kg/m²	50	2.90	(2.66)	2.38	(2.56)	2.26	(2.55)			
20–<25 kg/m²	147	2.41	(2.36)	1.82	(2.08)	1.92	(2.34)			
25–<30 kg/m²	72	2.26	(2.00)	1.97	(1.96)	1.49	(1.64)			
≥30 kg/m²	25	1.76	(1.71)	1.74	(1.79)	1.20	(1.71)			

M: Mean; SD: Standard deviation; Main effect: ANOVA effect of the independent variable listed in the left column; Time: effect of the timepoint (t1, t2, t3); Interaction: interaction between the independent variable and the factor timepoint.

**Table 6:** Presence of feeding tube and tracheostomy at t1–t3; - no, + yes.

	At t1 t2 t3	Number of Patients	Percent		At t1 t2 t3	Number of Patients	Percent
Feeding tube (- no, + yes)	- - -	165	60.4	Tracheostomy (- no, + yes)	- - -	184	66.4
	- - +	2	0.7		- + -	3	1.1
	- + -	2	0.7		- + +	6	2.2
	- + +	5	1.8		+ - -	29	10.5
	+ - -	39	14.3		+ - +	3	1.1
	+ + -	16	5.9		+ + -	10	3.6
	+ + +	44	16.1		+ + +	42	15.2

## Data Availability

The datasets presented in this article are not readily available because of patient confidentiality and participant privacy terms. Requests to access the datasets should be directed to the corresponding author.
